# Descending control of motor sequences in *Drosophila*

**DOI:** 10.1016/j.conb.2023.102822

**Published:** 2023-12-13

**Authors:** Julie H. Simpson

**Affiliations:** Dept. Molecular Cellular and Developmental Biology and Neuroscience Research Institute, University of California Santa Barbara, USA

## Abstract

The descending neurons connecting the fly’s brain to its ventral nerve cord respond to sensory stimuli and evoke motor programs of varying complexity. Anatomical characterization of the descending neurons and their synaptic connections suggests how these circuits organize movements, while optogenetic manipulation of their activity reveals what behaviors they can induce. Monitoring their responses to sensory stimuli or during behavior performance indicates what information they may encode. Recent advances in all three approaches make the descending neurons an excellent place to better understand the sensorimotor integration and transformation required for nervous systems to govern the motor sequences that constitute animal behavior.

## Introduction

Descending neurons (DNs) connect the fly’s brain to its ventral nerve cord (VNC). ~1300 DNs are responsible for transmitting sensory and contextual information from the brain (130,000 neurons) to enable detailed execution of motor programs in the VNC (22,000 neurons) [1–3]. Since relatively few DNs pass through the neck connective, they represent a substantially compressed encoding of information, both in terms of the incoming sensory stimuli they integrate and the outgoing behavior responses they elicit.

The organization and function of DNs have been investigated by anatomical characterization, optogenetic analysis of behavioral contributions, and recording of activity. Multiple lines of evidence support the view that, in general, the DNs convey higher-level commands, goals, or decisions about what behavior the fly should perform rather than detailed instructions about the individual actions that compose them. Categorizing the different morphological types of DNs showed that many are single bilateral pairs, while others are small groups with similar shapes and projections [1–3]. These types may reflect command-like and population-coding archetypes, but further causative behavioral and correlative activity measurements will illuminate how these two modes of organizing motor control co-exist.

This review attempts to summarize the recent explosion of research centered around descending neurons and motor control, highlighting the complementarity of different experimental approaches and the value of comparisons among datasets, illustrated by advances in our understanding of the neural control mechanisms for producing more complex behavioral sequences such as courtship and grooming. While most of the work to date has focused on the contributions of DNs to motor circuits, some may affect sensory processing or provide modulatory cues as well. Great progress has been made, but ample room for surprising discoveries remains. Activating combinations of DNs with patterned stimuli, quantifying limb movements with greater resolution, and recording from multiple DNs simultaneously during behaviors, will be needed to assess the full range of potential codes carried by this numerically limited set of neurons.

### Anatomical organization of descending neurons

Cross-sections of the neck connective in various fly species, using electron microscopy, showed approximately 3000 neuron profiles, which include both ascending and descending neurons. Dye-filling neurons in the neck and mapping those with cell body locations in the brain, coupled with reagents to assign neurotransmitter identity, led to an estimate of 1100 DNs representing cholinergic, GABAergic, glutamatergic, and aminergic types [4]. This estimate is close to the 1328 DNs counted in the single animal sectioned to produce the MANC connectome [2].

In order to assess how many different functional types of DN might exist, it was useful to ask if each DN is anatomically distinct or if there are sets of similar cells. Some of the DNs are immediately recognizable. For instance, the pair of giant fibers implicated in the jump escape response are recognizable landmarks with huge axon diameters. A large-scale effort to generate genetic reagents to target populations of DNs enabled characterization of the morphology of 350–500 pairs [1], covering approximately 29% of the total number of DNs [2]. Recent electron microscopy (EM) datasets and connectome analyses for the adult fly brain [5–8] and female and male ventral nerve cord [9,10] have identified 376 additional types [2]. The rarest class is single, unique neurons with bilateral projections, reminiscent of aminergic neurons from earlier in development or modulatory neurons associated with conveying contextual state [11]. More DNs occur as morphologically specialized, bilaterally symmetric pairs, with neurites of varying complexity. An appealing hypothesis is that these single pairs are early-born primary neurons establishing axon guidance pathways and foundational functional capacities of the adult animal; most of the experimentally identified command-like neurons described below belong to this type. Other DNs occur in small sets (2–15 neurons/hemisphere) of anatomically similar cells. These may represent population codes, where the number of neurons recruited titrate the intensity of the behavior. Alternatively, subtle differences in their connectivity may permit them to regulate related but distinct functions.

DN nomenclature is coordinated with the rest of the central nervous system [12,13] and based on cell body location, axon tract membership, and VNC target neuropil [1–3,8]. By definition, “descending neurons” receive synaptic inputs in the brain and make synaptic outputs in the ventral nerve cord. Many DNs also make output synapses in the brain, especially in the suboesophageal zone (SEZ), a region in the ventral anterior part of the brain with developmental and evolutionary affiliations to the VNC [14] that has been implicated in a range of sensory and motor functions [15]. Many DNs receive additional synaptic input (onto their axons) from local VNC neurons [2]. DNs also connect to each other [16,17]. Considering partners with more than 10 synapses, a given DN may receive inputs from tens to hundreds of other neurons and send outputs to similar numbers. Individual DNs have different ratios of input and output synapses overall in the brain and VNC. The computational capacity of individual DNs is just beginning to be investigated [18].

The most logical current proposal for functional organization of the DNs is related to their role in motor control. While dendrites of DNs are found in many brain regions, their axons tend to innervate discrete regions of the VNC [1]. The ones targeting the ventral leg neuropils may coordinate walking and grooming; those synapsing into the dorsal tectulum (wing and haltere) may be involved with flight; while others that span multiple VNC regions potentially coordinate leg and wing movements for behaviors involving different appendages, such as escape or courtship [1,2,19]. Connectome analysis in the female shows 209 DNs connect to the (left) legs and 272 to the (left) wing regions [20]; numbers in the male are similar [2]. The significance of the variability in connected partners and synapse numbers should become clearer as more individual samples are analyzed by EM and light microscopy (see below), but some trends are apparent. For example, while some DNs synapse onto motor neurons directly, this accounts for less than 10% of their output [2]. Most DNs influence movement indirectly, via synapses onto pre-motor circuits. Although there are as many as 70 motor neurons (MNs) per leg [9,21], there are thousands of VNC local interneurons associated with each leg, which suggests that the implementation of rhythmic patterns and inter-joint, inter-leg coordination will be both flexible and complex [2,20]. Local premotor neurons are more likely to synapse onto motor neurons innervating a common module (leg segment and muscle type), while DNs can synapse onto motor and premotor neurons belonging to different functional units [2]. Combinatorial, sequential activation of motor neurons could be achieved by synaptic weighting within premotor neurons or by DNs connecting to multiple premotor circuits. The lack of frequent direct DN to MN synapses suggests that DNs probably do not form motor synergies (groups of muscles that work together) themselves but might recruit them into sequences or modify the rhythmic timing of their activity through DN to pre-motor circuit connections. Further investigation into the architecture of DN synaptic outputs–which groups of premotor neurons they target and which sets of motor neurons and muscles those ultimately effect–will guide the behavioral experiments to test these predictions of DN contributions.

To derive insights into DN function or organization from their anatomy, the spatial resolution of electron microscopy for synaptic connectivity, and the ability to assess reproducibility by confocal microscopy in multiple replicates where the same neurons are genetically targeted, make a complementary combination. The EM connectome work was especially challenging because the current datasets from the brain and VNC were collected separately, requiring manual matching of neurons across the divide using landmarks or diagnostic neurite morphology guided by the light-level confocal microscopy images (DN-splitGAL4 lines and multi-color flip-out clones). A complete nervous system, where the neck connective between the brain and ventral nerve cord is intact, is eagerly awaited to help corroborate these deductive identity assignments as well as assess biological variability. And while these anatomical resources provide new insights and powerful resources, our current understanding of neural circuit organization is heavily based on sparser functional studies.

### Behavioral changes caused by perturbations of descending neuron activity

In the overarching goal of determining how neural circuits coordinate animal behavior, descending neurons represent an anatomically critical and experimentally tractable control point. The limited behavioral capacities of decapitated flies hint at what descending neurons normally do. A fly without its head displays the righting reflex: if knocked over, it can still coordinate its limbs sufficiently to stand up. Decapitated flies do not walk but can take a few steps if prodded, and they will groom in response to bristle deflections, dust, or the application of certain drugs to the neck connective [22–24]. Thermogenetic activation of some developmental lineages of neurons induces coherent behaviors in decapitated flies, ranging from wing grooming or flapping to jumping, but activation of other lineages causes disorganized or uncoordinated leg movements [25]. These observations indicate that while basic sensory-motor reflexes can be performed by neural circuits located entirely in the VNC [26], more complicated and coordinated behaviors require the sensory apparatus on the head, control neurons in the brain, and the descending neurons connecting them to the VNC.

A survey of the capacity of descending neurons to elicit behavioral programs comes from optogenetic activation screens [27] using splitGAL4 lines [1,28]. Constant redlight stimulation was provided to groups of adult flies expressing *UAS-csChrimson* [29] in different DN populations, and a range of behaviors were observed, including walking and grooming. Different DNs elicited similar behaviors, suggesting some redundancy. The same neurons could evoke different behaviors depending on what the fly was doing right before activation (context), indicating that neurons can have different effects [27], perhaps depending on the combination of other active neurons.

The following examples highlight DNs with command-like functions, but this may represent experimental design or selection bias: neurons whose activation evokes recognizable behaviors are dramatic. Additionally, some descending neurons that were identified in activation screens as capable of inducing behaviors do not show obvious silencing phenotypes [30]. This may argue for redundancy, where other neurons can also convey the signals essential for normal performance of the behavior [2], or context dependence, where particular environmental conditions are also required to display an effect. Alternatively, we may not be measuring behavioral changes with sufficient resolution to determine unique contributions.

**Figure 1:**
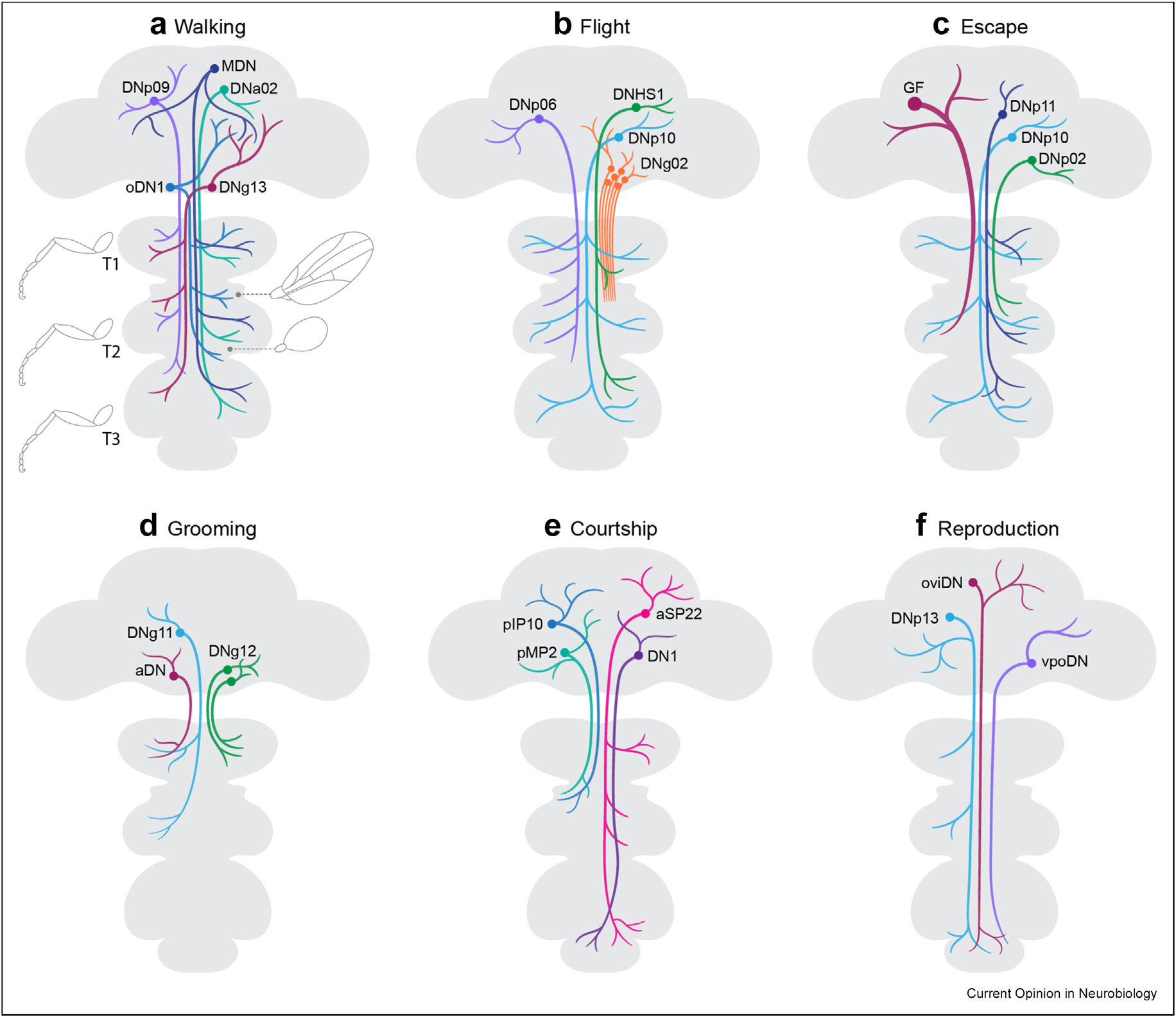
Schematic of descending neurons contributing to different behaviors. Examples are shown of individual identified neurons or groups with similar morphology that can command behaviors or control specific movements. The outline of the nervous system and the leg and wing drawings were modeled on a figure from Cheong et al., 2023, and show which VNC segmental neuromeres are associated with the sensory and motor circuits for each appendage. *Figure by Elfy Chiang, VisLab, MRC Laboratory of Molecular Biology*. a. Walking: **DNp09** – freeze [62] OR forward walk with ipsilateral turns during courtship [36]; **MDN** – backward walking [31]; **oDN1** – bolt forward walking [37]; **DNa02** and **DNg13** – steering [39,90] b. Flight: **DNg02** – steering [47]; **DNHS1** (DNp15) – looming [53]; **DNp06** – maintaining straight flight [52]; **DNp10** – landing initiation [45]. c. Escape: giant fiber (**GF**; DNp01) – escape [96]; **DNp10** – jump [45]; **DNp11** and **DNp02** jump [2]; d. Grooming: **aDN** – antennal grooming [71]; **DNg11** – front leg rubbing and **DNg12** anterior grooming [73]. e. Courtship: **pIP10** – song [78]; **pMP2** – song [97] and type [79,80]; **pSP22** – proboscis extension, abdominal bending [81]; **DN1** – olfactory-initiated chasing [77]. f. Reproduction: **OviDN** – oviposition [86]; **vpoDN** – acceptance [82]; **DNp13** – rejection [83,84]. VNC, ventral nerve cord

Some descending neurons induce very specific phenotypes (Figure 1). For example, activation of Moonwalker DNs makes flies walk backward [31]. Visual and olfactory cues processed by sensory circuits in the brain [32,33], and tactile cues from the forelegs carried by ascending neurons to the brain [34], trigger the Moonwalker neurons, which are required for flies to back up normally when they reach a dead end. Moonwalker’s postsynaptic targets showed that the precise sequence of muscle and joint movements needed to step backward are elicited by separate premotor neurons [35]. Thus, Moonwalker DNs are command-like, activated by the integration of sensory cues in the brain, and transmitting a signal to perform a motor program whose details are executed by distributed motor control circuits located in the VNC.

What about walking forward? Is that one behavior or many? An animal might need to walk in a variety of contexts: searching for food, approaching a mate, avoiding a threat, and exploring new territory. Parallel DNs may convey the command to walk under different circumstances, or the range of drives may converge onto a shared circuit, triggering a common motor program. The DNp09 DNs integrate visual cues to mediate pursuit during courtship, while the BPN neurons in the brain connect to other DNs to elicit fast, straight walking [36], arguing for some parallel pathways dedicated to walking for specific purposes. New evidence indicates that there may be distinct circuits that control stopping in different contexts as well [37].

Descending circuits may be required to balance or skew the properties of left and right steps to achieve a directional heading. Since simultaneous manipulation of bilateral pairs of neurons might be expected to promote starts or stops, identification of DNs that control turns requires a different experimental strategy. Detecting differences in neuronal activity between left and right homologs correlated with steering led to the implication of DNa01 and DNa02 [38]: these neurons initiate a common motor command, whether the impetus is an acute sensory cue or a remembered directional goal, illustrating convergence. DNs do not always evoke higher-order behavioral goals. An interesting study of DNs during visually guided steering in walking flies showed activity correlated with specific leg movements and at precise timepoints during the step cycle, suggesting much more fine-grained control [39]. Excellent recent reviews of motor control and sensory feedback in locomotion [40–42] provide additional perspectives.

The probability and speed of walking can be modulated by serotoninergic neurons in the VNC [43], and there are aminergic DNs as well [4]. Dopaminergic DNs promote both locomotion and grooming [22,44], while octopamine has been implicated in flight [45]. Aminergic DNs may modify motor commands by regulating activation thresholds or setting the context for which motor programs are available. Do DNs modulate sensory inputs? Possibly. Connectivity analysis shows that some DNs do synapse onto sensory rather than premotor circuits, and a recent report shows that peptidergic allatostatin-positive Epione DNs can suppress behavioral responses to noxious heat, but the VNC neurons through which these EpiDNs act are still unknown [46].

These examples emphasize the behavioral effects of unique, command-like neurons; evidence for a population code model is rarer. A recent study of descending neurons involved in flight describes the DNg02 neuron type, which encompasses 15 neurons with similar morphology [47]. They connect quite directly between the visual motion circuits and the motor neurons innervating flight muscles. Activation of DNg02 neurons in flying flies increases the wingbeat amplitude, and the more DNg02 neurons that are activated, the greater the increase, resembling a size-principle effect [48] in which both neuron number and activation level contribute to maximum power output. These DNg02 neurons respond to large-field visual motion, and left and right can respond separately, perhaps to balance thrust and maintain straight flight [49]. Silencing DNg02s disrupts the optomotor reflex [50]. Although these neurons are morphologically similar, further assessment of their connectivity reveals some differences. Since these DNs were identified by activating them when the fly was flying, they may contribute to other behaviors in other contexts. Thus, whether DNg02s reflect a population coding for a shared function or similar but distinct visuomotor transforms remains to be seen. The population of DNg02 works with other flight-descending neurons implicated in steering (AX) [51], evasive turns (DNp06) [52], or encoding self-motion (DNOVS1, DNOVS2, DNHS1) [53]. New results identify DNs that can induce rapid saccade turns or straight flight, with an inhibitory neuron located in the brain arbitrating between them [54].

Escape behavior, where a fly responds to an expanding visual stimulus with a jump take-off into flight, is a classic example of a behavioral sequence elicited by descending command neurons [55]. Fast, directionless takeoff is triggered by the activation of the giant fiber neurons (DNp01). These two large-diameter axons are readily visible in the cross-sections of the neck connective in many insects [2,56,57]. They make both chemical and electrical synapses and both direct and indirect connections to motor neurons in the middle leg neuropils needed for leg extension at launch. Once the giant fibers reach their activation threshold, they evoke jump escape [58]. If, however, the visual looming stimulus expands more slowly, perhaps indicating a less urgent threat, an alternative pathway is recruited, and the fly takes off more gracefully, repositioning its legs and jumping away from the approaching object [58,59]. One of the most exciting hypotheses generated from recent connectome analysis is the proposal of candidates for this parallel descending pathway among the LTctDNs that target the region of the VNC spanning the leg and wing neuropils [2]. The short-mode and long-mode escape takeoffs illustrate competing motor sequences initiated in response to similar visual stimuli [60,61] and triggered by parallel descending circuits with different activation thresholds [58].

Escape is not always the best choice. Depending on what a fly’s neighbors are doing and whether the fly itself is still or already moving, the best defensive strategy against an aerial predator might be to freeze instead of flee. This is another example where a DN (DNp09) represents a bridge between sensory integration and motor execution, and its activation instantiates the decision to run or halt [62].

The descending neurons have been a rich vein for exploring mechanisms of context-dependent behavior selection [63]. Looming visual stimuli can activate the motor program for leg extension, which can be used for escape take-off… or for landing! The difference in the fly’s circumstances–earthbound standing or airborne flying–can be encoded by visual and proprioceptive cues that then influence the activity of specific descending neurons DNp07 or DNp10. Ache et al. [45] discovered descending neurons critical for appropriate landing behavior. Silencing them impairs landing, and activating them induces landing. Interestingly, their spike rate determines leg extension amplitude, implying that descending neurons are capable of more than an on-off contribution to this action selection.

### Links to larval locomotion

What about descending neurons in larvae? EM connectome analysis identifies 184 DNs connecting the brain to the SEZ and 182 DNs targeting the VNC (of 3016 total neurons) [17]. Although larval and adult flies have very different modes of locomotion, the moonwalker descending neurons (MDNs) are present in both life stages and induce backward movement in both [64,65]. This persistence of neuron and function seems unusual; moonwalker is the only case described in the literature so far, and the nervous system has been extensively remodeled through development [42]. Another neuron, PDM-DN, is an archetypical DN in that it integrates sensory information about changing olfactory signals to induce an appropriate motor sequence of stopping and turning as the larva navigates toward an odor source [66]. A recent analysis of whole brain connectomics in the first instar larva includes a catalog of the anatomy and brain connections of the larval descending neurons [17], including an intriguing “zig-zag” connection between PDM-DN and MDN via an ascending neuron; additional functional studies are eagerly awaited. Larvae also use descending neurons to control escape. Contragoro DNs synapse onto the command-like Goro neuron in the larval VNC that induces rolling escape behavior [67]. The convergence of nociceptive sensory information that triggers escape happens in both the larval brain and ventral nerve cord. Recent work identified peptidergic DNs that dampen the thermal pain response by inhibiting Goro neurons [68], modulating motor circuits rather than sensory ones. But sensory circuits can also be modulated: descending inhibitory neurons may gate the pain response during starvation to sustain feeding [69].

### More complex sequences

Behaviors can be subdivided into action sequences of different timescales and complexity. Walking can be decomposed into stance and swing phases, or even further into ordered changes in the angles of individual leg joints [40]. In escape, the fly repositions its legs, lifts its wings, and extends the middle legs to jump [70]. Grooming and courtship behaviors, described below, provide examples of motor sequences of increasing complexity, and ongoing experiments unveil the roles of DNs in coordinating them.

Fly grooming is a sequential combination of motor programs where the legs move in different patterns to remove debris. Descending neurons contribute to its hierarchical control. Command-like DNs for antennal cleaning [71], wing cleaning [72], head cleaning, and front leg rubbing [73] have been identified in activation screens, where they trigger ordered leg muscle contractions that produce targeted grooming movements. Since optogenetic activation of DNs with constant light results in temporally patterned muscle activation, the dynamic structure must be imposed by central pattern-generating circuits in between, feedback loops, or intrinsic electrical properties of the DNs themselves (such as refractory periods). While most of these DNs induce single behavioral subroutines, others induce combinations. Activation of DNg12, for example, evokes the alternation of head sweeps and front leg rubs that constitute anterior grooming, indicating that DNs are capable of inducing complex, goal-directed motor sequences. Current data does not yet address whether DN activation represents the normal decision point about which grooming subroutine to execute when multiple body parts are dirty. Experimental coactivation of DNs results in an alternation of the actions they elicit, indicating that conflicting commands can be resolved by neural circuits in the VNC, but inhibitory neurons connecting sensory inputs to DNs associated with competing actions suggest that the activation of a particular DN may represent the normal decision mechanism [73]. Simultaneous recording from different DNs during behavioral choices will be required to determine if activation of only one represents the physiological mechanism of action selection.

Descending neurons that contribute to reproductive behaviors provide an additional illustration of how complex sensory-motor sequences can be controlled. The neural circuitry that underlies male courtship behavior has been extensively dissected, starting with the seminal discovery of the *fruitless* mutant and the neurons that express the transcription factor that gene encodes (reviewed in Ref. [74]). Multiple sensory modalities converge to initiate courtship, and the accumulation of excitation induces progressive commitment via a sequence of motor programs from chasing through singing and proboscis contact to copulation (reviewed in Refs. [75,76]). Sensory integration with past social experience is performed in the brain, while coordination of the body and limbs requires the VNC, so as might be expected, DNs are key contributors. A male fly detects an appropriate female by her appearance, movement, and pheromones. The P1 neurons in the brain integrate these sensory inputs and arbitrate between courtship and aggression. There are DNs that receive synapses from the pheromone-detection circuit to initiate chasing [77]. Subsequent courtship songs can be induced by activation of the DN pIP10 [78]; sine and pulse elements of the song can be initiated by overlapping but distinct circuits [79]. Intriguingly, different descending neurons seem to govern the decision to sing and the selection of sine vs. pulse song [80], subdividing modular motor control in surprising ways. Other sequential steps in courtship have a common control point: proboscis extension, abdominal lifting, and attempted copulation occur in response to increased firing of the aSP22 DNs [81]. A single DN can elicit several motor programs in order because different postsynaptic targets are recruited as its firing rate increases.

A virgin female fly can accept a male’s courtship advances by opening her vaginal plates to permit copulation, while a mated female may extrude her ovipositor to reject him. These mutually exclusive motor programs are triggered by the same sensory cues interpreted under different mating state conditions. This information is integrated to activate the appropriate DN: vpoDN for acceptance [82] or DNp13 for rejection [83,84]. A choice is made in the brain and executed by selective activation of a DN (reviewed in Ref. [85]).

The ovipositor is also employed in egg laying, using a motor program that involves some of the same muscles as the rejection response but is controlled by different DNs. In a mated female, the OviDNs that control egg-laying (or oviposition) also integrate sensory cues about the appropriate substrate with the mating state [86]. The state is communicated when these neurons are disinhibited by sex peptide signaling that occurs during sperm transfer via pC1 neurons [86]. The pC1 neurons act as a decision-making center where female flies choose between aggression [87], receptivity to courtship, and egg-laying responses based on internal state and external context. The OviDNs accumulate sensory information over time, and when they reach an excitation threshold, their activation initiates the motor sequence for egg laying [88]. OviDN may encode the overarching command, but sensory and motor feedback contribute to the functional flexibility of the execution of its component steps [89].

Lessons about how descending neurons contribute to the control of motor sequences from courtship and reproductive behaviors include that DNs represent the integration point of sensory and state information, achieve activation thresholds, and tend to initiate or modulate rather than implement detailed motor programs. Temporal or spatial integration may be needed to induce DN activity. In some cases, a DN’s spike pattern is critical, but in others, the fact of their activation may be sufficient to instigate behavior. Courtship, like grooming, can be decomposed into simpler motor programs. Different DNs evoke different courtship subroutines: the neurons that respond to pheromones, drive song, or trigger proboscis extension are distinct. The full courtship sequence includes all of these actions, employed in order but with flexibility in timing and duration that may be enabled by the modularity of descending control and the ability of sensory feedback to tune aspects of sequence execution, as has now been demonstrated for egg-laying.

### Measurements of activity in descending neurons during behavior

Interrogating what sensory or contextual information descending neurons respond to requires measuring their activity while providing stimuli; determining which DN activity patterns might be causal requires simultaneously recording the fly’s behavior. Electrophysiological recordings from neurons in the neck connective showed that activity in dopaminergic DNs was correlated with walking [44]. However, activating or inhibiting these DNs did not alter leg movements [44]. Measuring and manipulating neural activity in specific populations of neurons in different environmental contexts and internal states while recording fly behavior will be required to fully assess the coding potential of descending neurons for sensory input and motor output.

In a recent technical tour-de-force, the Ramdya lab developed a dissection and imaging preparation to perform two-photon-based imaging of changes in GCaMP fluorescence, indicating calcium levels commonly thought to correlate with neural activity, in the neck connective and ventral nerve cord of a behaving fly [90]. The tethering required for stable imaging somewhat limits the range of activity-to-behavior correlations that can be assessed, but there is clear potential for discovery, as shown by their recordings of populations of DNs during spontaneous and odor-evoked behaviors [91]. The overall conclusion from this study is that a large fraction of DNs show activity changes during each behavior–and that this activity correlates with higher-order behavioral features (e.g. walking as a whole rather than extension of a particular leg segment). Whether the DNs activity is causing or responding to these behavioral states is not resolved, and the sensory stimuli the fly encountered–other than odor– are undetermined. A new study using the same preparation revealed a surprising degree of connectivity among descending neurons, suggesting that activation of one command-like neuron may recruit other DNs, resulting in a population code [16].

Combinations of neurons may act together to encode sensory inputs or modulate motor outputs. The ability to record from groups of neurons simultaneously–with suitable temporal resolution–will enable researchers to compare the timing of their activity: does whichever command-like DN responds first dictate the behavioral program selected? Do DNs cooperate to produce motor sequences by firing in order? New methods for imaging neural activity in freely-behaving flies, currently employed for the brain [92–94], may be applicable in the descending neurons to expand the range of behaviors and contexts in which correlations can be assessed. Virtual reality experiments, where sensory inputs can be precisely controlled, may also be informative [95]. We need to test what DNs can encode (show activity responses to) by presenting specific combinations of sensory and contextual information in the functional imaging preparation (ideally while also measuring what behaviors the fly performs in response). These correlations will complement the optogenetic experiments where DNs are activated to determine the motor programs they can induce. Together, recording neural activity assays information encoding while inducing neural activity assays motor control capacity, and we will need both to determine how DNs contribute to the normal organization of behavior.

## Conclusions

One of the critical jobs of the nervous system is to detect sensory and situational information–*do you smell food and are you hungry?* Then the detection circuits must integrate this information and determine an appropriate behavioral response: *yes, both; let’s eat*. The right sequence of movements should be initiated and executed: *pick up the fork, skewer the tomato, open the mouth, chew, and swallow*. Descending neurons connecting the brain to the ventral nervous system sit at the convergence point, responding to sensory cues and initiating motor programs. They are not passive relays, conveying unfiltered information, but rather specialized for integration. To understand how they do their jobs, we must learn what sensory cues they respond to and what actions they cause. Do the DNs encode the higher-level command “Eat!” or the detail-oriented sequence of actions required to accomplish consumption? And are these orders carried by single neurons or population codes? These debates will likely be resolved by the discovery that both situations coexist. Their computational potential may be extended if we find that the same descending neuron can induce different actions when fired in different temporal patterns, in different environmental contexts, or in conjunction with different peers. Complete anatomical description of DN types and their connectivity, combined with expanding genetic access for functional imaging and optogenetic manipulation, coupled with simultaneous quantitative behavioral assessment, make the *Drosophila* descending neurons a powerful experimental system to decipher how different parts of the nervous system communicate, and specifically how sensory and contextual information is compressed to initiate higher-order commands that then must be decompressed to execute the complex motor sequences we recognize as behavior.

## Data Availability

No data was used for the research described in the article.
